# Switching from tipranavir (TPV) 500/ritonavir (RTV) 200 mg to TPV 500/RTV 100 mg in treatment-experienced patients (pts) with HIV RNA <50 copies/mL

**DOI:** 10.1186/1758-2652-13-S4-P49

**Published:** 2010-11-08

**Authors:** JL Casado, P Domingo, R Rubio, A Antela, MA Lopez-Ruz, A Castro, J Portilla, E Ribera, D Podzamczer, JA Oteo, J Galindo, S Otero, F Lozano, V Estrada, J Moltó, S Moreno

**Affiliations:** 1 Hospital Ramón y Cajal, Madrid, Spain; 2 Hospital Sant Pau, Barcelona, Spain; 3 Hospital 12 de Octubre, Madrid, Spain; 4 Hospital Clinico, Santiago De Compostela, Spain; 5 Hospital Virgen de las Nieves, Infectious Diseases, Granada, Spain; 6 Hospital Juan Canalejo, A CORUÑA, Spain; 7 Hospital Geneal, ALICANTE, Spain; 8 Hospital Vall d'Hebron, Barcelona, Spain; 9 Hospital de Bellvitge, Barcelona, Spain; 10 Hospital de La Rioja, Logroño, Spain; 11 Hospital Clínico, Valencia, Spain; 12 Hospital de Calella, Barcelona, Spain; 13 Hospital de Valme, Sevilla, Spain; 14 Hospital Clinico San Carlos, Madrid, Spain; 15 Hospital Germans Trias i Pujol, Badalona, Spain

## Background

The administration of 200 mg of RTV to boost TPV plasma levels is associated with poor tolerance and toxicity. Some studies have shown that 100 mg of RTV could be enough to reach plasma levels of TPV that inhibit most HIV strains. This clinical trial was designed to show the efficacy and tolerability of switching to TPV 500/RTV 100 in patients with suppressed viremia receiving TPV 500/RTV 200.

## Methods

Open, randomized, multicenter clinical trial. Pts who were receiving TPV 500/RTV 200 with an HIV RNA<50 copies/mL for at least 6 months and with a genotypic sensitivity score showing activity of TPV were randomized to continue on the same dose or to switch to TPV 500/RTV 100 mg. The efficacy endpoint was the proportion of pts with HIV RNA<50 copies/mL at 48 weeks in an ITT analysis. Discontinuation due to intolerance, liver toxicity and lipid abnormalities were also evaluated. TPV plasma trough levels were measure in all the patients.

## Results

35 pts were randomized: 16 pts continued on TPV 500/RTV 200 mg while 19 pts were switched to the lower TPV 500/RTV 100 mg dose. At baseline, 48% had AIDS, and mean CD4 count was 515 cells/mm3. Coinfection with HCV was more frequent in pts who continued on the 200 mg RTV group (44%) than in those that switched to the 100 mg RTV group (11%)(p=0.02). Pts had received a median of 10 drugs (including 3.6 PI), and mean duration of TPV therapy was 32 months. Mean number of accompanying drugs was 3.4 in the two groups. Mutations in RT and PRO before initiating therapy with TPV were found in 31% and 20% pts, respectively. After 48 weeks, HIV RNA remained below 50 copies/mL in 89.5% pts in the RTV100 group and in 75% in the RTV200 group [ITT, difference 14.5% (95%CI -10.5, 39.5%)]. Mean CD4 count change was +24 in the RTV100 group, and -33 in the RTV200 group (p=0.5). A greater decrease in GPT, total cholesterol, and triglycerides was observed in the RTV100 group. One pt on the lower dose developed grade 2 increase in total cholesterol, while 3 pts developed grade 2-4 toxicities in the higher dose group (grade 2 and 4 hypertriglyceridemia, grade 2 hypertransaminemia). Median TPV trough levels at 48 weeks were 38854 ng/mL and 25465 ng/mL in the RTV 200 and RTV 100 groups, respectively.

## Conclusions

Switching from TPV 500/RTV 200 mg to TPV 500/RTV 100 mg in treatment-experienced patients with HIV RNA <50 copies/mL maintains virological suppression, while improving liver and lipid abnormalities.

